# Acute Bouts of Exercising Improved Mood, Rumination and Social Interaction in Inpatients With Mental Disorders

**DOI:** 10.3389/fpsyg.2018.00249

**Published:** 2018-03-13

**Authors:** Serge Brand, Flora Colledge, Sebastian Ludyga, Raphael Emmenegger, Nadeem Kalak, Dena Sadeghi Bahmani, Edith Holsboer-Trachsler, Uwe Pühse, Markus Gerber

**Affiliations:** ^1^Division of Sport Science and Psychosocial Health, Department of Sport, Exercise and Health, University of Basel, Basel, Switzerland; ^2^Center for Affective, Stress and Sleep Disorders, Psychiatric Clinics, University of Basel, Basel, Switzerland; ^3^Substance Abuse Prevention Research Center and Sleep Disorders Research Center, Kermanshah University of Medical Sciences, Kermanshah, Iran

**Keywords:** physical activity, mental disorders, mood, rumination, *trans*-diagnostic

## Abstract

**Background:** Studies at the macro level (such as longer-term interventions) showed that physical activity impacts positively on cognitive-emotional processes of patients with mental disorders. However, research focusing on the immediate impact of acute bouts of exercise (micro level) are missing. The aim of the present study was therefore to investigate whether and to what extent single bouts of moderately intense exercise can influence dimensions of psychological functioning in inpatients with mental disorders.

**Method:** 129 inpatients (mean age: 38.16 years; 50.4% females) took part and completed a questionnaire both immediately before and immediately after exercising. Thirty inpatients completed the questionnaires a second time in the same week. The questionnaire covered socio-demographic and illness-related information. Further, the questionnaire asked about current psychological states such as mood, rumination, social interactions, and attention, tiredness, and physical strengths as a proxy of physiological states.

**Results:** Psychological states improved from pre- to post-session. Improvements were observed for mood, social interactions, attention, and physical strengths. Likewise, rumination and tiredness decreased. Mood, rumination, and tiredness further improved, when patients completed the questionnaires the second time in the same week.

**Conclusion:** At micro level, single bouts of exercise impacted positively on cognitive-emotional processes such as mood, rumination, attention and social interactions, and physiological states of tiredness and physical strengths among inpatients with mental disorders. In addition, further improvements were observed, if patients participated in physical activities a second time.

## Introduction

There is mounting evidence that physical inactivity has become the new ‘smoking’; in other words, physical inactivity has become a serious health concern at an epidemiologic level. High inactivity scores are associated with higher early mortality rates (Ekelund et al., 2016), along with huge economic costs both for the individual and for the public authorities (Ding et al., 2016).

While physical inactivity is a serious health concern for a larger part of the population (Booth et al., 2012), this observation holds particularly true for patients with mental disorders: Systematic reviews and meta-analyses (Rosenbaum et al., 2014; Schuch et al., 2016; Mikkelsen et al., 2017) have shown that low rates of physical activity are observed among patients with a broad variety of mental disorders. Along with these low rates of physical activity, patients with mental disorders are at increased risk of diabetes mellitus type II (Roy and Lloyd, 2012; Lloyd et al., 2015), metabolic syndrome (McElroy and Keck, 2014; Vancampfort et al., 2015b), or increased tobacco use disorder (McMahon, 1999; Williams and Ziedonis, 2004; Baker et al., 2007; Aubin et al., 2012; Mendelsohn et al., 2015; Stepankova et al., 2016). Specifically, Bartoli et al. (2015) in their meta-analysis showed that patients with schizoaffective disorders had an increased risk to suffer from metabolic syndrome, compared to patients with non-affective psychosis and schizophrenia. Similarly, Tang et al. (2017) showed in their meta-analysis an association between the occurrence of anxiety disorders and the risk of suffering from metabolic syndrome.

On the other hand, meta-analyses and systematic reviews support the notion that “exercise as medicine” (Pedersen and Saltin, 2015; Gerber et al., 2016; Hallgren et al., 2016) also benefits patients with mental disorders such as major depressive disorders (Knapen et al., 2015; Stubbs et al., 2016; Bailey et al., 2017; Krogh et al., 2017; Schuch et al., 2017; Sukhato et al., 2017; Vancampfort et al., 2017a; Wu et al., 2017), including among elderly patients (Mikkelsen et al., 2017; Perez-Lopez et al., 2017), post-partum depression (McCurdy et al., 2017; Poyatos-Leon et al., 2017; Pritchett et al., 2017; Saligheh et al., 2017), bipolar disorders (Vancampfort et al., 2017a), post-traumatic stress disorders (Rosenbaum et al., 2015; Vancampfort et al., 2016), anxiety disorders (Gordon et al., 2017; Stubbs et al., 2017; Vancampfort et al., 2017b,c,d), and psychosis/schizophrenia (Pajonk et al., 2010; Keller-Varady et al., 2017; Mittal et al., 2017; Tarpada and Morris, 2017; Vancampfort et al., 2017a), while among patients with substance use disorders the evidence supporting regular exercise is weaker [alcohol use disorder: (Vancampfort et al., 2015a; Hallgren et al., 2017); tobacco use disorder: (Ussher et al., 2014)].

Thus, while there is extant research on the longer-term impact of regular exercise training (macro level) on psychological functioning in patients with mental disorders, surprisingly, research on the influence of single bouts of exercise (micro level) in this population is scarce: Meyer et al. (2016) (see also below) investigated the influence of a single bout on mood among female patients with major depressive disorders, and single bouts of exercise were associated with enhanced executive functions among pediatric patients with attention-deficit/hyperactivity disorders (ADHD) (Ludyga et al., 2017). Similarly, Gerber et al. (2013) found that single bouts of aerobic exercise in burn-out patients were linked to an increase in positive mood states, and a decrease in negative mood states.

Meyer et al. (2016) tested the difference of self-selected vs. prescribed exercise intensity on mood change among female patients with major depressive disorders, and observed that, contrary to expectations, mood increased after a single bout of exercise at prescribed intensity, compared to a self-selected exercise intensity. This result was surprising, as previous research on healthy participants showed that self-selected exercise intensity was associated with increased feeling of pleasure and motivation to exercise again (Ekkekakis, 2009; Ekkekakis et al., 2008, 2011; Lind et al., 2008). In their first experiment Bernstein and McNally (2017a) showed that among a sample of healthy young adults, after a negative mood-inducing intervention, negative feelings persisted, but to a lesser degree, if prior to the negative mood-induction a single bout of moderate aerobic exercise was performed. Likewise, Bernstein and McNally (2017b) showed that a single bout of moderate aerobic exercise was associated with a higher capacity to overcome negative feelings. Taking the results of Meyer et al. (2016) and Bernstein and McNally (2017a,b) into account, the aim of this study is to address the issue of whether, and to what extent, mood will increase after a single bout of exercising among inpatients with mental disorders. In line with this, we expanded upon previous research in that we assessed inpatients with mental disorders under naturalistic everyday hospital settings.

Research on psychological functioning among patients with mental disorders has showed that rumination is a predictor for the emergence and maintenance of dysfunctional thoughts. Following Nolen-Hoeksema et al. (1999) and Shors et al. (2017), rumination is understood as the constant repetition of thoughts, often impairing more constructive and goal-oriented thoughts. Typically, among patients with mental disorders, rumination is associated with autobiographical and, most dramatically, negative content. Soo et al. (2009) showed among patients with chronic illness that rumination predicted the emergence and maintenance of emotional distress. Likewise, Kertz et al. (2015) showed that a higher extent of rumination predicted poorer outcomes of a brief psychotherapeutic intervention in patients with depressive and anxiety disorders. Law and Tucker (2017) claimed that rumination was associated with feelings of entrapment and hopelessness, which, in the most severe cases, may contribute to the onset of suicidal ideation and then facilitate the transition from thinking about suicide to a suicide attempt. Further, given that rumination occurs in various mental disorders such as depressive and anxiety disorders (McEvoy et al., 2013; Spinhoven et al., 2015), Law and Tucker (2017) claimed that rumination is considered *trans*-diagnostic, that is to say: rumination is a cognitive-emotional process observed within a broad variety of mental disorders, including patients with insomnia: see Norton for an extensive overview of the *trans*-diagnostic approach (Norton et al., 2013, 2015; Norton and Paulus, 2016; Norton and Roberge, 2017; Pearl and Norton, 2017). We took both rumination and the *trans*-diagnostic approach into account in choosing to assess the degree to which a single bout of exercising might favorably impact on rumination among patients with mental disorders, irrespective of their psychiatric diagnoses.

Next, there is extant literature showing that at least among healthy samples and pediatric patients with ADHD a single bout of exercise improved executive function and attention [see Ludyga et al. (2016, 2017) for a broader overview], while such an intervention has not so far been trialed among adult inpatients with mental disorders. Accordingly, a further goal was to investigate whether a single bout of exercise improved self-rated attention among patients with mental disorders.

Further, by nature, exercise improves physical strength and reduces tiredness. For instance, in a pilot-study with 12 male burnout patients, Gerber et al. (2013) showed that single bouts of exercise were associated with decreased fatigue, whereas patients reported higher perceived activation after having been engaged in aerobic exercise training. Accordingly, these two dimensions were further introduced as outcome variables.

Lastly, compared to people without mental disorders, people with mental disorders had a tremendously increased risk of reporting psychosocial impairments and difficulties (Munjiza et al., 2014; Jobst et al., 2015); however, a survey of psychiatric hospitals revealed that inpatients expected to increase their social contacts while attending sports and exercise sessions (Brand et al., 2016). In line with this, Masten et al. (2011) showed that neurophysiological responses to peer rejection appeared to be a biomarker for the onset of adolescents’ risk for depression. Therefore, the next aim of the present study was to investigate whether, and to what extent, a single bout of exercise might favorably influence their views on social interaction.

Collectively, at a macro level, results from meta-analyses and systematic reviews have suggested that physical activity interventions have the potential to favorably impact on psychological processes among patients with mental disorders. By contrast, little research has focused on the benefits associated with physical exercise from a micro-level perspective. Although benefits following a single bout are considered transient, they may allow a fast improvement in psychological functioning. The present study aimed at bridging this gap in the literature. Specifically, we expanded upon previous research in that (1) unlike Meyer et al. (2016) and Bernstein and McNally (2017a,b) we did not assess participants under laboratory circumstances, but under naturalistic conditions; (2) we focused on a broad variety of cognitive-emotional information processing such as mood, rumination, and attention; (3) we did not group patients depending on their mental disorders [Meyer et al. (2016): female outpatients with major depressive disorders; meta-analyses and systematic reviews focusing on patients with specific mental disorders such as major depressive disorders, post-partum depression, bipolar disorders, PTSD, anxiety disorders, psychosis/schizophrenia or substance use disorders; see above]; rather, we followed the approach suggested by Norton et al. (2013, 2015), Norton and Paulus (2016), Norton and Roberge (2017), Pearl and Norton (2017) and employed a *trans*-diagnostic approach; (4) based on recent results on the beneficial effect of regular exercising outdoor (Lawton et al., 2017), we asked, if a singly bout of outdoor exercising led to more favorable effects compared to a single bout of indoor exercising; (5) we asked if a beneficial effect of exercising might be observable, when participants attended a session for a second time. We hold that the present study has the potential to enhance the understanding of the influence of single bouts of exercise on the cognitive-emotional processing of patients with a broad variety of mental disorders, based on a protocol feasibly in everyday conditions of the psychiatric hospital.

## Materials and Methods

### Procedure

From September 2016 to March 2017, inpatients from the Psychiatric Hospital of the University of Basel (UPK, Universitäre Psychiatrische Kliniken Basel, Basel, Switzerland) were approached during their participation in different physical activity interventions (see below) and asked to complete a short questionnaire (see below) immediately before and immediately after a physical activity session. Participants were informed about the aims of the study and the secure and anonymous data handling, and signed the written informed consent sheet. Further, and always on a voluntary basis, patients were encouraged to completing the questionnaires also for a further exercising session. The study was approved by the local ethics committee (Ethikkommission Nordwestschweiz, EKNZ, Basel, Switzerland), and performed in accordance with the ethical principles laid down in the Declaration of Helsinki and its later amendments.

### Sample

Collectively, 129 inpatients took part in the study. Inclusion criteria were: (1) age between 18 and 65 years; (2) inpatients of the psychiatric hospital; (3) sufficient reading and writing skills to complete the German questionnaire; (4) signed written informed consent.

### Physical Activity Sessions

Patients attended the following physical activity courses: Nordic walking: *n* = 56 inpatients (43.41%); workout/gymnastics: *n* = 49 inpatients (38%); ball sports: *n* = 14 inpatients (11%); *n* = 10 inpatients (7.8%) did not provide further information. Single sessions lasted for 40–60 min, and exercise training was performed at moderate intensity. Except Nordic Walking, all other activities were indoor activities.

### Questionnaire

We employed a self-administered questionnaire.

The first part covered socio-demographic (gender and age) and illness-related [diagnosis, if known, duration of illness (years), duration of stay in hospital (days)] information.

The second part addressed the type of physical activity engaged in. Participants ticked a box for: Nordic walking, jogging, workout, endurance training; dancing (folkloric dancing; ‘jazz-dance’); ball sports; others.

The third part asked about current affective states and cognitions: Mood: “Currently, my mood is…” [6-points Likert scale from 1 (=very low) to 6 (=very high); test–retest-reliability: *r* = 0.84]; Rumination: “Currently, I’m always mulling over the same problems” [6-points Likert scale from 1 (=completely true) to 6 (=not at all true); test–retest-reliability: *r* = 0.73]; Tiredness: “Currently, I feel…” [6-points Likert scale from 1 (=very tired) to 6 (=not at all tired); test–retest-reliability: *r* = 0.71]; Physical strengths: “Currently, I feel …” [6-points Likert scale from 1 (=very flat) to 6 (=very energetic); test–retest-reliability: *r* = 0.74]; Attention: “Currently, I’m able to focus and to keep track of a task such as reading the newspaper, completing forms, following a conversation, watching a movie, remembering my daily duties, etc.” [6-points Likert scale from 1 (=not at all true) to 6 (=completely true); test–retest-reliability: *r* = 0.88]; Importance of social interaction: “Currently, I appreciate being in touch with other people [6-points Likert scale from 1 (=not at all true) to 6 (=completely true); test–retest-reliability: *r* = 0.72].

### Statistical Analysis

Pre- to post comparisons were performed with a series of *t*-tests for related samples. Effect sizes were reported with the following ranges: *d* = 0–0.19: trivial [T]; *d* = 0.20–0.49: small [S]; *d* = 0.50–0.79: medium [M]; *d* = 0.80 and greater: large [L].

To compare, if compared to indoor exercising, outdoor Nordic Walking had a stronger effect on Mood, Rumination, Tiredness, Physical Strengths, Attention, and Social interaction, a series of single *t*-tests were performed.

To compare changes over two sessions, ANOVAs for repeated measures were performed, with Time (4 time points) and Group (patients with one session vs. patients with two sessions) as factors, and dimensions of Mood, Rumination, Tiredness, Physical Strengths, Attention, and Social interaction as dependent variables. To do so, we employed the intent-to-treat (ITT) algorithm with the last observation carried forward (LOCF), this is to say: For those patients with only one session, the identical values were implemented to the second session. Effect sizes were indicated with the partial eta squared ($\eta_{p}^{2}$), with 0.059 ≥ $\eta_{p}^{2}$ ≥ 0.01 indicating small [S], 0.139 ≥ $\eta_{p}^{2}$ ≥ 0.06 indicating medium [M], and $\eta_{p}^{2}$ ≥ 0.14 indicating large [L] effect sizes.

All statistics were performed with SPSS^®^ 25.0 (IBM Corporation, Armonk, NY, United States) for Apple^®^ Mac^®^.

## Results

### Sample Characteristics

Collectively, 129 inpatients (mean age: *M* = 38.89, *SD* = 13.89; 50.4% females) took part in the study. They self-reported the following main psychiatric diagnoses: major depressive disorders: *n* = 89 inpatients (69%); substance use disorder: *n* = 14 inpatients (10,9%); bipolar disorders: *n* = 3 inpatients (2.3%); anxiety disorders: *n* = 2 inpatients (9.3%); no information: *n* = 11 inpatients (8.5%). Mean stay in hospital was 23 days (*SD* = 10.45). Stay at hospital (days) and duration of illness (years) did not statistically differ between the self-reported diagnoses (*F* < 1, *p* > 0.4). All patients were under specific psychotherapeutic and psychopharmacologic treatment at therapeutic dosages.

### Pre- to Post-assessment for One Session

Descriptive and inferential statistical indices are reported in **Table 1**.

**Table 1:** Descriptive and inferential statistical indices of the first session.

			Time points		
		
		Pre-assessment	Post-assessment	*t*-tests	*d*
Mood	“Currently, my mood is…”	3.56 (1.25)	4.63 (1.25)	*t*(127) = 6.82^∗∗∗^	0.64 [M]
Rumination	“Currently, I’m always mulling over the same problems”	3.26 (1.57)	4.61 (1.37)	*t*(127) = 4.05^∗∗∗^	0.92 [L]
Tiredness	“Currently, I feel …” [tired vs. alert]	3.13 (1.52)	3.99 (1.46)	*t*(127) = 3.95^∗∗∗^	0.59 [M]
Physical strengths	“Currently, I feel …” [flat vs. energetic]	3.24 (1.34)	3.89 (1.37)	*t*(127) = 3.08^∗∗∗^	0.47 [S]
Attention	“Currently, I’m able to focus and to keep track”	3.08 (1.27)	4.13 (1.26)	*t*(125) = 3.87^∗∗∗^	0.87 [L]
Social interaction	“Currently, I appreciate being in touch with other people”	3.24 (1.67)	4.32 (1.39)	*t*(125) = 2.85^∗∗^	0.71 [M]


**^∗∗^p < 0.01, ^∗∗∗^p < 0.001. [S], small effect size; [M], medium effect size; [L], large effect size.**

Physical strength significantly increased from pre-to post-testing, though the effect size was small (*d* = 0.47).

Mood, Tiredness, and Social interaction from pre- to post-assessment, and effect sizes were medium (*d* = 0.50–0.79)

Rumination and Attention significantly improved from pre- to post-assessment, and effect sizes were large (*d* = 0.80 and greater).

### Differences Between Outdoor (Nordic Walking) and Indoor Exercising

Neither at the beginning, nor at the end of the first session Mood, Rumination, Tiredness, Physical Strengths, Attention, and Social interaction did statistically differ between participants exercising outdoors (Nordic Walking group: *n* = 56), compared to participants exercising indoors (*n* = 67; all *t*’s < 1.3, *p*’s > 0.35).

### Changes of Psychological Dimensions Over Two Sessions

Patients, who completed the questionnaires during two sessions in the same week, did not descriptively and statistically differ from those patients, who completed the questionnaires just once during an exercising session as regards age, gender, self-reported psychiatric diagnosis, and duration of stay in the hospital (all *t*’s < 1; *p*s < 0.23).

All descriptive and inferential statistical indices are reported in **Tables 2**, **3**.

**Table 2:** Descriptive statistical indices of dimensions of mood, rumination, tiredness, physical strengths, attention, and social interaction, for two time points and separately for patients with one and with two assessments.

		One session (*N* = 99)		Two sessions (*N* = 30)	
			
		Pre-assessment	Post-assessment	Pre-assessment	Post-assessment
**(A) Time point 1**
Mood	“Currently, my mood is…”	3.66 (1.40)	4.36 (1.21)	3.87 (1.32)	4.35 (1.26)
Rumination	“Currently, I’m always mulling over the same problems”	3.76 (1.58)	4.18 (1.38)	3.20 (1.49)	4.10 (1.79)
Tiredness	“Currently, I feel being…” [tired vs. alert]	3.26 (1.52)	3.79 (1.49)	3.28 (1.69)	3.97 (1.43)
Physical strengths	“Currently, I feel being…” [flat vs. energetic]	3.43 (1.34)	3.87 (1.39)	3.76 (1.22)	3.86 (1.33)
Attention	“Currently, I’m able to focus and to keep track”	3.93 (1.50)	4.08 (1.52)	4.50 (1.19)	4.20 (1.24)
Social interaction	“Currently, I do appreciate being in touch with other people”	3.76 (1.68)	4.25 (1.40)	4.10 (1.73)	4.55 (1.43)
**(B) Time point 2**
Mood	“Currently, my mood is…”	3.66 (1.40)	4.36 (1.21)	3.77 (0.85)	5.12 (0.86)
Rumination	“Currently, I’m always mulling over the same problems”	3.76 (1.58)	4.18 (1.38)	4.18 (1.38)	4.93 (0.91)
Tiredness	“Currently, I feel being…” [tired vs. alert]	3.26 (1.52)	3.79 (1.49)	3.55 (1.21)	4.34 (0.94)
Physical strengths	“Currently, I feel being…” [flat vs. energetic]	3.43 (1.34)	3.87 (1.39)	3.34 (1.52)	3.93 (1.39)
Attention	“Currently, I’m able to focus and to keep track”	3.93 (1.50)	4.08 (1.52)	3.57 (1.04)	4.63 (1.07)
Social interaction	“Currently, I do appreciate being in touch with other people”	3.76 (1.68)	4.25 (1.40)	3.68 (1.18)	4.76 (1.24)


**Table 3:** Inferential statistical indices of dimensions of mood, motivation, rumination, motivation/curiosity, tiredness, physical strengths, attention, and social interaction, for two time points and separately for patients with one and with two assessments.

			Statistics		
		
		Time *F* $\eta_{p}^{2}$	Group *F* $\eta_{p}^{2}$	Time × Group interaction *F* $\eta_{p}^{2}$	Greenhouse-Geisser Epsilon
Mood	“Currently, my mood is…”	53.18^∗∗∗^ 0.320 [L]	1.49 0.013 [S]	7.26^∗∗∗^ 0.060 [S]	0.463
Rumination	“Currently, I’m always mulling over the same problems”	16.53^∗∗∗^ 0.188 [M]	0.02 0.000 [S]	6.06^∗∗^ 0.045 [S]	0.713
Tiredness	“Currently, I feel being…” [tired vs. alert]	19.61^∗∗∗^ 0.140 [L]	3.61 0.029 [S]	9.87^∗∗∗^ 0.112 [M]	0.708
Physical strengths	“Currently, I feel being…” [flat vs. energetic]	10.64^∗∗∗^ 0.080 [M]	0.22 0.002 [S]	0.73 0.006 [S]	0.529
Attention	“Currently, I’m able to focus and to keep track”	19.22^∗∗∗^ 0.133 [M]	1.83 0.014 [S]	2.19 0.017 [S]	0.731
Social interaction	“Currently, I do appreciate being in touch with other people”	21.26^∗∗∗^ 0.151 [L]	2.97 0.032 [S]	0.25 0.002 [S]	0.662


*Factors are: Time, Group, and the Time by Group interaction.*

*Degrees of freedom: Time; Group by Group interaction: (3, 375); Group: (1, 125). ^∗^ = 0.05, ^∗∗^ = 0.01, ^∗∗∗^ =0.001. [S], small effect size; [M], medium effect size; [L], large effect size.*

**FIGURE 1:**
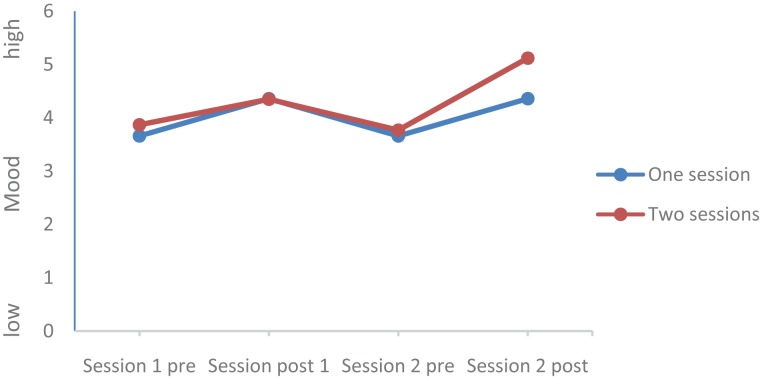
Mood.

**FIGURE 2:**
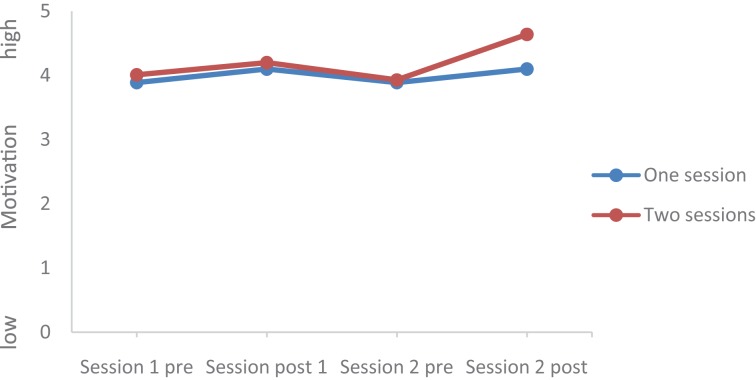
Motivation.

**FIGURE 3:**
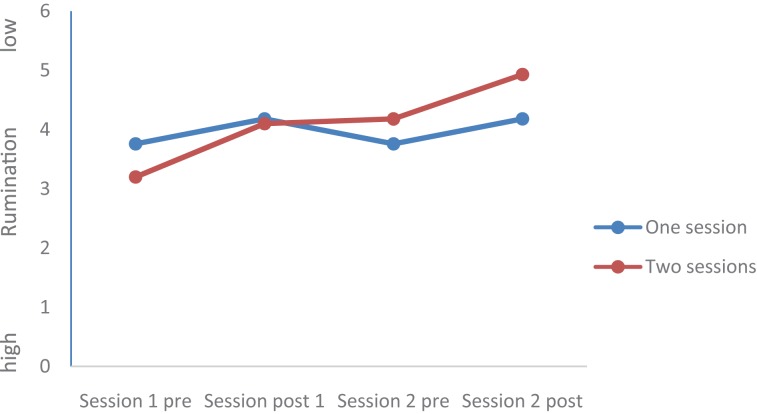
Rumination.

**FIGURE 4:**
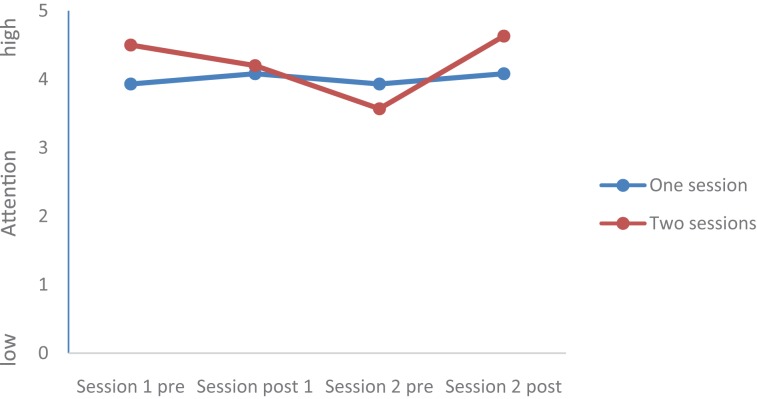
Attention.

**FIGURE 5:**
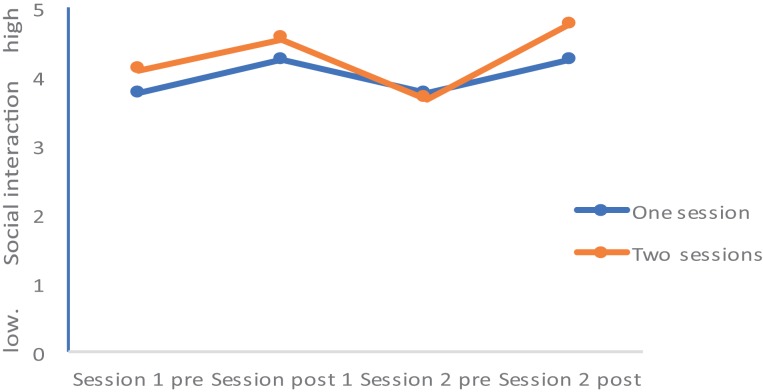
Social interaction.

Values of all dimensions (Mood, Rumination, Tiredness, Physical strengths, Attention, and Social Interaction) changed over time; Group (completers of one session vs. completers of two sessions) did not statistically differ, and the Time by Group interactions were significant for Mood, Rumination, and Tiredness, while the Time by Group interactions were not significant for Physical Strengths, Attention, and Social interaction. *Post hoc* tests after Bonferroni–Holm for corrections for *p*-values (0.05/7 = 0.0071; 0.05/6 = 0.0083; 0.05/5 = 0.01; 0.05/4 = 0.0125; 0.05/3 = 0.016; 0.05/2 = 0.025; 0.05/1 = 0.05) showed that at the end of the second session, values of the completers of the second session were statistically and significantly more favorable compared to the values of the completers of only one session for the following dimensions: Mood (see **Figures 1**–**5**), Tiredness, Rumination, and Attention. No statistically significant mean differences were observed for Physical strengths and Social interaction.

## Discussion

The key findings of the present study were that after a single bout of exercise, inpatients with mental disorders reported improvements in mood, physical strength, attention, and appreciation of social interaction. At the same time, rumination and tiredness decreased. Further, over two sessions, the mentioned pattern of results did further change in a favorable direction, suggesting therefore a constancy in changes as a result of single bouts of exercise. The present results add to the current literature in an important way, in that among inpatients with mental disorders small, but significant improvement in psychological dimensions at micro level, that is, after a single session of exercise, could be demonstrated. Finally, we employed the *trans*-diagnostic approach, and showed that improvements were observed irrespective of the self-reported psychiatric diagnoses.

While the pattern of results is promising, the quality of the data does not allow a deeper understanding of the underlying psychological or physiological mechanisms. For want of direct evidence, we propose the following explanations drawn from other research.

As regards increased mood, some studies have shown that acute exercise activity improves mood. To illustrate, Meyer et al. (2016) showed that after single exercise bouts mood increased among female patients with major depressive disorders. Likewise, Bernstein and McNally (2017a,b) showed that recovery from negative mood was easier and faster if prior moderate physical activity occurred. However, we expanded upon previous research in that we addressed the issue of mood enhancement not only under laboratory conditions, but in naturalistic settings of a psychiatric clinic, and, most importantly, employing a *trans*-diagnostic approach. We hold that the present study conditions more accurately mirror everyday life settings of psychiatric hospitals, and therefore allow a more authentic transfer of the present data and study design to other psychiatric hospitals.

However, the observation that mood improved after a single bout of exercise does not explain the mechanism behind this improvement.

This holds particularly true as regards the zero-differences of the comparisons between outdoor (Nordic Walking) and indoor activities: Participants of the Nordic Walking sessions (outdoor activity) did report neither more favorable (nor more unfavorable) outcome scores, compared to participants of the indoor physical activities. This pattern of results in contrast to the findings of Lawton et al. (2017): They assessed a sample of healthy participants who met the UK recommendations of regular physical activity guidelines. Further, participants completed a questionnaire to assess relatedness to nature. Lawton et al. (2017) showed that participants who engaged in outdoor physical activities were also those participants, who reported significantly lower somatic anxiety levels and higher scores of relatedness to nature. However, we also note that the present results are comparable only to a limited extent with the those of Lawton et al. (2017), as methodological differences (samples, interventions, questionnaires, health status, duration, frequency, intensity, and type of physical activity) appeared to preclude a more thorough comparison and conclusion.

From a neuropsychological point of view, Sartori et al. (2011) and Salehi et al. (2016) showed that regular exercise training improved both symptoms of depression and BDNF levels. While it might be therefore conceivable that mood improved via increased BDNF, Salehi et al. (2016) also showed that both improved mood and BDNF increase were statistically completely unrelated, pointing to a physiology-psychology miss-match. Ekkekakis et al. (2008, 2011) and Ekkekakis (2009) argued that physical activity *per se* might be an agreeable sensation, which, by nature, might lead to feelings of well-being and improved mood. From a psychological point of view, Knapen et al. (2005, 2015) argued that physical activity has the power to improve self-esteem, self-efficacy, feelings of pride and achievement, and stress-resistance [see also Gerber et al. (2015a,b)]. While Knapen et al. (2005, 2015) attributed such improvements to longer lasting interventions, in the present study we were able to show that improved mood was observable after a single bout of exercise among inpatients with a broad variety of psychiatric diagnoses.

As regards attention, several overviews (Ludyga et al., 2016) showed that a single bout of moderate exercise improved selective attention in healthy participants, and in pediatric patients with ADHD (Ludyga et al., 2017). We claim that the present findings are in accord with previous research, though, again, we have added to the current literature in assessing self-reported attention among inpatients with a broad range of self-reported mental disorders, thus taking into account the *trans*-diagnostic approach.

In our opinion, improvements in rumination after a single bout of exercise deserves special attention: By definition, rumination is considered dysfunctional, as it impairs a more straightforward and goal-oriented thinking; accordingly, reducing rumination should be considered a small but important step in patients’ modification of cognitive-emotional concepts (Ahmadpanah et al., 2017a,b). However, again, the quality of the data does not allow a deeper understanding of the underlying cognitive-emotional processes. Though highly speculative, we suggest the following explanations: Following others (Eysenck et al., 2007; Ahmadpanah et al., 2016), the capacity of the working memory is limited, and in patients with anxiety disorders it appears that their working memory is overloaded by cognitive-emotional processes related to anxiety and coping with anxiety. Such a working memory overload is most probably also observable among a broad range of patients with mental disorders. We suggest that engagement in physical activity and exercise demand working memory and cognitive-emotional processes, and consequently there are fewer resources to devote to rumination. While such a theoretical approach cannot be tested with the present data, future studies might use dual task paradigms (Plummer et al., 2015; Manicolo et al., 2017) to investigate the extent to which physical activity might decrease rumination. However, we also hold that the present study adds to the current literature in that we showed that a single bout of exercise favorably impacted on rumination in inpatients with mental disorders.

Next, in our opinion, the improvement of tiredness might be best explained by an increased psychophysiological arousal following exercising. Actually, such decreases in tiredness has been observed among patients with bourn-out after single bouts of exercise; accordingly, patients reported higher perceived activation after having been engaged in aerobic exercise training (Gerber et al., 2013). Likewise, and to a further extent, patients with multiple sclerosis reported lower scores of depression and fatigue after an 8-week intervention of yoga and aquatic exercising, compared to a control condition (Razazian et al., 2016).

Next, data showed that single bouts of exercise had a positive impact on social interactions. While again the quality of the data does not allow a broader understanding, we suggest that similar to the mechanisms underlying the decrease of rumination, social interactions were perceived as attractive, or less problematic, due to the modifications of the load on the working memory. Alternatively, one might also claim that an increased interest in social interaction is a mere by-product and epiphenomenon of increased mood, decreased rumination, and higher attention. Irrespective of a possible explanation, we believe that the present results are important, as patients with mental disorders have a highly increased risk of reporting unsatisfying and aversive social interactions (Madsen et al., 2009; Munjiza et al., 2014; Teicher and Samson, 2016).

Last, for the following reasons, the improvements after the second session are impressive: First, it appears that the favorable changes follow a linear improvement (**Tables 2**, **3** and **Figures 1**–**5**). Second, statistically significant changes were observed for Mood, Rumination, Tiredness, and Attention. Improvements in mood (Meyer et al., 2016; Bernstein and McNally, 2017a,b) and attention (Ludyga et al., 2016, 2017) after single bouts of exercising were already previously reported. The present results expand upon these findings, in that among patients with mental disorders improvements in mood, rumination, tiredness and attention after two sessions within 1 week have not been described so far. While again the quality of the data does not allow a data-driven explanation, very speculatively, one might follow Ekkekakis (2009), Ekkekakis et al. (2008, 2011), and Lind et al. (2008), who observed that increased feelings of pleasure and motivation did increase the odds to exercising again. Likewise, one might claim that such psychological mechanisms might have occurred also in those participants attending exercise courses and completing the questionnaires twice the same week.

Collectively, the present results suggest that for patients with mental disorders physical activity participation twice the week led to subjectively assessed favorable outcomes.

Despite these novel and promising results, several limitations warrant against overgeneralization of the findings. First, we fully relied on self-reported data; while such an approach is suitable for psychological dimensions, self-reported psychiatric diagnoses could have been double-checked with the official diagnoses of experts. However, the following reasons motivated us to forego to experts’ diagnoses: First, we wanted to keep the entire assessment and patients’ work load as easy as possible. Second, patients’ adherence to the study might have further decreased, if further personnel were involved. Third, we followed a *trans*-diagnostic approach; accordingly, we were convinced that changes would have occurred irrespective of psychiatric diagnoses. The second limitation is that the sample might have been biased in three ways: First, most probably due to the autumn and winter season, only a small group participated on the outdoor Nordic Walking program, while a recent research showed the beneficial effect of outdoor regular physical exercising on anxiety and well-being (Lawton et al., 2017). Second, only patients willing and able to following the exercise interventions were included. Third, only patients willing and able to complete the questionnaire were assessed. The last ‘negative’ selection bias is particularly critical, as only 30 of 129 patients were willing to complete the questionnaires for a second session, and as from a previous survey (Brand et al., 2016), it turned out that only about 25% of patients in psychiatric hospitals regularly participated in the broad variety of sport and exercise activities offered, while further 25% of inpatients participated in these programs on an irregular basis. Thus, because participation in physical activity and exercise programs is voluntary, it is conceivable that most inpatients assessed in the present study were motivated to engage in exercise activities. It is therefore unclear whether the same positive effects on current affective states and cognitions would have occurred in less motivated inpatients. Third, no psychophysiological data were assessed, though biomarkers such as BDNF, cortisol, CCK, or objective sleep parameters might have allowed a deeper understanding of the psychobiological processes. In line with this, fourth, the present pattern of results might have emerged due to further unassessed dimensions, which might have biased two or more dimensions in the same or opposite directions. Fifth, characteristics of the physical activity such as duration, intensity, frequency, and cardiorespiratory load were not systematically assessed, either subjectively or objectively. Thus, we cannot offer insights into which activity types and which exercise modalities were most efficient in triggering positive emotional and cognitive responses.

## Conclusion

Single bouts of physical exercise impacted positively on psychological dimensions of patients hospitalized with a variety of mental disorders. Further, the pattern of results remained stable when a subsample of inpatients completed the questionnaires a second time. The favorable changes at the micro level might explain the favorable changes of psychological dimensions at the macro level. Further, at a methodological level, visualizing such small but significant changes in current affective states and cognitions might help to improve inpatients’ commitment toward exercise, and motivate them to maintain a physically active lifestyle in the long run.

## Author Contributions

SB, FC, SL, RE, NK, DSB, EH-T, UP, and MG wrote the proposal and designed the study and completed the final draft. SB, FC, RE, NK, DSB, and MG were involved in data gathering and data entering. SB, FC, SL, NK, DSB, and MG performed the statistical analysis and wrote the draft. EH-T, NK, and UP commented on the second draft. All authors commented on the final manuscript, which was completed by SB, FC, SL, NK, DSB, EH-T, UP, and MG.

## Conflict of Interest Statement

The authors declare that the research was conducted in the absence of any commercial or financial relationships that could be construed as a potential conflict of interest.

## References

[B1] Ahmadpanah M. Akbari T. Akhondi A. Haghighi M. Jahangard L. Sadeghi Bahmani D. (2017a). Detached mindfulness reduced both depression and anxiety in elderly women with major depressive disorders. *Psychiatry Res.* 257 87–94. 10.1016/j.psychres.2017.07.030 28735173

[B2] Ahmadpanah M. Astinsadaf S. Akhondi A. Haghighi M. Sadeghi Bahmani D. Nazaribadie M. (2017b). Early maladaptive schemas of emotional deprivation, social isolation, shame and abandonment are related to a history of suicide attempts among patients with major depressive disorders. *Compr. Psychiatry* 77 71–79. 10.1016/j.comppsych.2017.05.008 28636896

[B3] Ahmadpanah M. Keshavarz M. Haghighi M. Jahangard L. Bajoghli H. Sadeghi Bahmani D. (2016). Higher emotional intelligence is related to lower test anxiety among students. *Neuropsychiatric Disease Treat.* 12 133–136. 10.2147/NDT.S98259 26834474 PMC4716726

[B4] Aubin H. J. Rollema H. Svensson T. H. Winterer G. (2012). Smoking, quitting, and psychiatric disease: a review. *Neurosci. Biobehav. Rev.* 36 271–284. 10.1016/j.neubiorev.2011.06.007 21723317

[B5] Bailey A. P. Hetrick S. E. Rosenbaum S. Purcell R. Parker A. G. (2017). Treating depression with physical activity in adolescents and young adults: a systematic review and meta-analysis of randomised controlled trials. *Psychol. Med.* 10.1017/S0033291717002653 [Epub ahead of print]. 28994355

[B6] Baker A. Richmond R. Haile M. Lewin T. J. Carr V. J. Taylor R. L. (2007). Characteristics of smokers with a psychotic disorder and implications for smoking interventions. *Psychiatry Res.* 150 141–152. 10.1016/j.psychres.2006.05.021 17289155

[B7] Bartoli F. Crocamo C. Caslini M. Clerici M. Carra G. (2015). Schizoaffective disorder and metabolic syndrome: a meta-analytic comparison with schizophrenia and other non-affective psychoses. *J. Psychiatr. Res.* 66–67, 127–134. 10.1016/j.jpsychires.2015.04.028 26004300

[B8] Bernstein E. E. McNally R. J. (2017a). Acute aerobic exercise hastens emotional recovery from a subsequent stressor. *Health Psychol.* 36 560–567. 10.1037/hea0000482 28277695

[B9] Bernstein E. E. McNally R. J. (2017b). Acute aerobic exercise helps overcome emotion regulation deficits. *Cognit. Emot.* 31 834–843. 10.1080/02699931.2016.1168284 27043051

[B10] Booth F. W. Roberts C. K. Laye M. J. (2012). Lack of exercise is a major cause of chronic diseases. *Compr. Physiol.* 2 1143–1211. 10.1002/cphy.c110025 23798298 PMC4241367

[B11] Brand S. Colledge F. Beeler N. Puhse U. Kalak N. Sadeghi Bahmani D. (2016). The current state of physical activity and exercise programs in German-speaking, Swiss psychiatric hospitals: results from a brief online survey. *Neuropsychiatric Dis. Treat.* 12 1309–1317. 10.2147/NDT.S107313 27350748 PMC4902243

[B12] Ding D. Lawson K. D. Kolbe-Alexander T. L. Finkelstein E. A. Katzmarzyk P. T. van Mechelen W. (2016). The economic burden of physical inactivity: a global analysis of major non-communicable diseases. *Lancet* 388 1311–1324. 10.1016/S0140-6736(16)30383-X 27475266

[B13] Ekelund U. Steene-Johannessen J. Brown W. J. Fagerland M. W. Owen N. Powell K. E. (2016). Does physical activity attenuate, or even eliminate, the detrimental association of sitting time with mortality? A harmonised meta-analysis of data from more than 1 million men and women. *Lancet* 388 1302–1310. 10.1016/S0140-6736(16)30370-1 27475271

[B14] Ekkekakis P. (2009). Let them roam free? Physiological and psychological evidence for the potential of self-selected exercise intensity in public health. *Sports Med.* 39 857–888. 10.2165/11315210-000000000-00000 19757863

[B15] Ekkekakis P. Hall E. E. Petruzzello S. J. (2008). The relationship between exercise intensity and affective responses demystified: to crack the 40-year-old nut, replace the 40-year-old nutcracker! *Ann. Behav. Med.* 35 136–149. 10.1007/s12160-008-9025-z 18369689

[B16] Ekkekakis P. Parfitt G. Petruzzello S. J. (2011). The pleasure and displeasure people feel when they exercise at different intensities: decennial update and progress towards a tripartite rationale for exercise intensity prescription. *Sports Med.* 41 641–671. 10.2165/11590680-000000000-00000 21780850

[B17] Eysenck M. W. Derakshan N. Santos R. Calvo M. G. (2007). Anxiety and cognitive performance: attentional control theory. *Emotion* 7 336–353. 10.1037/1528-3542.7.2.336 17516812

[B18] Gerber M. Brand S. Elliot C. Holsboer-Trachsler E. Puhse U. Beck J. (2013). Aerobic exercise training and burnout: a pilot study with male participants suffering from burnout. *BMC Res. Notes* 6:78. 10.1186/1756-0500-6-78 23497731 PMC3599602

[B19] Gerber M. Holsboer-Trachsler E. Puhse U. Brand S. (2016). Exercise is medicine for patients with major depressive disorders: but only if the “pill” is taken! *Neuropsychiatric Dis. Treat.* 12 1977–1981. 10.2147/NDT.S110656 27540294 PMC4981216

[B20] Gerber M. Jonsdottir I. H. Arvidson E. Lindwall M. Lindegard A. (2015a). Promoting graded exercise as a part of multimodal treatment in patients diagnosed with stress-related exhaustion. *J. Clin. Nurs.* 24 1904–1915. 10.1111/jocn.12820 25939917

[B21] Gerber M. Lindwall M. Brand S. Lang C. Elliot C. Puhse U. (2015b). Longitudinal relationships between perceived stress, exercise self-regulation and exercise involvement among physically active adolescents. *J. Sports Sci.* 33 369–380. 10.1080/02640414.2014.946072 25098842

[B22] Gordon B. R. McDowell C. P. Lyons M. Herring M. P. (2017). The effects of resistance exercise training on anxiety: a meta-analysis and meta-regression analysis of randomized controlled trials. *Sports Med.* 47 2521–2532. 10.1007/s40279-017-0769-0 28819746

[B23] Hallgren M. Vancampfort D. Giesen E. S. Lundin A. Stubbs B. (2017). Exercise as treatment for alcohol use disorders: systematic review and meta-analysis. *Br. J. Sports Med.* 51 1058–1064. 10.1136/bjsports-2016-096814 28087569

[B24] Hallgren M. Vancampfort D. Stubbs B. (2016). Exercise is medicine for depression: even when the “pill” is small. *Neuropsychiatr. Dis. Treat.* 12 2715–2721. 10.2147/NDT.S121782 27822043 PMC5087774

[B25] Jobst A. Sabass L. Palagyi A. Bauriedl-Schmidt C. Mauer M. C. Sarubin N. (2015). Effects of social exclusion on emotions and oxytocin and cortisol levels in patients with chronic depression. *J. Psychiatr. Res.* 60 170–177. 10.1016/j.jpsychires.2014.11.001 25466833

[B26] Keller-Varady K. Varady P. A. Roh A. Schmitt A. Falkai P. Hasan A. (2017). A systematic review of trials investigating strength training in schizophrenia spectrum disorders. *Schizophr. Res.* 10.1016/j.schres.2017.06.008 [Epub ahead of print]. 28602648

[B27] Kertz S. J. Koran J. Stevens K. T. Bjorgvinsson T. (2015). Repetitive negative thinking predicts depression and anxiety symptom improvement during brief cognitive behavioral therapy. *Behav. Res. Ther.* 68 54–63. 10.1016/j.brat.2015.03.006 25812825

[B28] Knapen J. Van de Vliet P. Van Coppenolle H. David A. Peuskens J. Pieters G. (2005). Comparison of changes in physical self-concept, global self-esteem, depression and anxiety following two different psychomotor therapy programs in nonpsychotic psychiatric inpatients. *Psychother. Psychosomat.* 74 353–361. 10.1159/000087782 16244511

[B29] Knapen J. Vancampfort D. Morien Y. Marchal Y. (2015). Exercise therapy improves both mental and physical health in patients with major depression. *Disabil. Rehabil.* 37 1490–1495. 10.3109/09638288.2014.972579 25342564

[B30] Krogh J. Hjorthoj C. Speyer H. Gluud C. Nordentoft M. (2017). Exercise for patients with major depression: a systematic review with meta-analysis and trial sequential analysis. *BMJ Open* 7:e014820. 10.1136/bmjopen-2016-014820 28928174 PMC5623558

[B31] Law K. C. Tucker R. P. (2017). Repetitive negative thinking and suicide: a burgeoning literature with need for further exploration. *Curr. Opin. Psychol.* 22 68–72. 10.1016/j.copsyc.2017.08.027 28888174

[B32] Lawton E. Brymer E. Clough P. Denovan A. (2017). The relationship between the physical activity environment, nature relatedness, anxiety, and the psychological well-being benefits of regular exercisers. *Front. Psychol.* 8:1058. 10.3389/fpsyg.2017.01058 28694788 PMC5483473

[B33] Lind E. Ekkekakis P. Vazou S. (2008). The affective impact of exercise intensity that slightly exceeds the preferred level: ‘pain’ for no additional ‘gain’. *J. Health Psychol.* 13 464–468. 10.1177/1359105308088517 18420754

[B34] Lloyd C. E. Sartorius N. Cimino L. C. Alvarez A. Guinzbourg de Braude M. Rabbani G. (2015). The INTERPRET-DD study of diabetes and depression: a protocol. *Diabet. Med.* 32 925–934. 10.1111/dme.12719 25659409

[B35] Ludyga S. Brand S. Gerber M. Weber P. Brotzmann M. Habibifar F. (2017). An event-related potential investigation of the acute effects of aerobic and coordinative exercise on inhibitory control in children with ADHD. *Dev. Cogn. Neurosci.* 28 21–28. 10.1016/j.dcn.2017.10.007 29100212 PMC6987879

[B36] Ludyga S. Gerber M. Brand S. Holsboer-Trachsler E. Puhse U. (2016). Acute effects of moderate aerobic exercise on specific aspects of executive function in different age and fitness groups: a meta-analysis. *Psychophysiology* 53 1611–1626. 10.1111/psyp.12736 27556572

[B37] Madsen K. A. McCulloch C. E. Crawford P. B. (2009). Parent modeling: perceptions of parents’ physical activity predict girls’ activity throughout adolescence. *J. Pediatr.* 154 278–283. 10.1016/j.jpeds.2008.07.044 18789455 PMC2654401

[B38] Manicolo O. Grob A. Hagmann-von Arx P. (2017). Gait in children with attention-deficit hyperactivity disorder in a dual-task paradigm. *Front. Psychol.* 8:34. 10.3389/fpsyg.2017.00034 28154547 PMC5243797

[B39] Masten C. L. Eisenberger N. I. Borofsky L. A. McNealy K. Pfeifer J. H. Dapretto M. (2011). Subgenual anterior cingulate responses to peer rejection: a marker of adolescents’ risk for depression. *Dev. Psychopathol.* 23 283–292. 10.1017/S0954579410000799 21262054 PMC3229829

[B40] McCurdy A. P. Boule N. G. Sivak A. Davenport M. H. (2017). Effects of exercise on mild-to-moderate depressive symptoms in the postpartum period: a meta-analysis. *Obstet. Gynecol.* 129 1087–1097. 10.1097/AOG.0000000000002053 28486363

[B41] McElroy S. L. Keck P. E. Jr. (2014). Metabolic syndrome in bipolar disorder: a review with a focus on bipolar depression. *J. Clin. Psychiatry* 75 46–61. 10.4088/JCP.13r08634 24502861

[B42] McEvoy P. M. Watson H. Watkins E. R. Nathan P. (2013). The relationship between worry, rumination, and comorbidity: evidence for repetitive negative thinking as a transdiagnostic construct. *J. Affect. Disord.* 151 313–320. 10.1016/j.jad.2013.06.014 23866301

[B43] McMahon R. J. (1999). Child and adolescent psychopathology as risk factors for subsequent tobacco use. *Nicotine Tob. Res.* 1(Suppl. 2), S45–S50; discussion S69–S70. 11768186 10.1080/14622299050011801

[B44] Mendelsohn C. P. Kirby D. P. Castle D. J. (2015). Smoking and mental illness. An update for psychiatrists. *Australas Psychiatry* 23 37–43. 10.1177/1039856214562076 25512967

[B45] Meyer J. D. Ellingson L. D. Koltyn K. F. Stegner A. J. Kim J. S. Cook D. B. (2016). Psychobiological responses to preferred and prescribed intensity exercise in major depressive disorder. *Med. Sci. Sports Exerc.* 48 2207–2215. 10.1249/MSS.0000000000001022 27387295

[B46] Mikkelsen K. Stojanovska L. Polenakovic M. Bosevski M. Apostolopoulos V. (2017). Exercise and mental health. *Maturitas* 106 48–56. 10.1016/j.maturitas.2017.09.003 29150166

[B47] Mittal V. A. Vargas T. Osborne K. J. Dean D. Gupta T. Ristanovic I. (2017). Exercise treatments for psychosis: a review. *Curr. Treat. Options psychiatry* 4 152–166. 10.1007/s40501-017-0112-2 29034144 PMC5636011

[B48] Munjiza J. Law V. Crawford M. J. (2014). Lasting personality pathology following exposure to catastrophic trauma in adults: systematic review. *Person. Mental Health* 8 320–336. 10.1002/pmh.1271 25123294

[B49] Nolen-Hoeksema S. Larson J. Grayson C. (1999). Explaining the gender difference in depressive symptoms. *J. Pers. Soc. Psychol.* 77 1061–1072. 10.1037/0022-3514.77.5.1061 10573880

[B50] Norton A. R. Abbott M. J. Norberg M. M. Hunt C. (2015). A systematic review of mindfulness and acceptance-based treatments for social anxiety disorder. *J. Clin. Psychol.* 71 283–301. 10.1002/jclp.22144 25529254

[B51] Norton P. J. Barrera T. L. Mathew A. R. Chamberlain L. D. Szafranski D. D. Reddy R. (2013). Effect of transdiagnostic cbt for anxiety disorders on comorbid diagnoses. *Depress. Anxiety* 30 168–173. 10.1002/da.22018 23212696

[B52] Norton P. J. Paulus D. J. (2016). Toward a unified treatment for emotional disorders: update on the science and practice. *Behav. Ther.* 47 854–868. 10.1016/j.beth.2015.07.002 27993337

[B53] Norton P. J. Roberge P. (2017). Transdiagnostic therapy. *Psychiatric Clin. North Am.* 40 675–687. 10.1016/j.psc.2017.08.003 29080593

[B54] Pajonk F. G. Wobrock T. Gruber O. Scherk H. Berner D. Kaizl I. (2010). Hippocampal plasticity in response to exercise in schizophrenia. *Arch. Gen. Psychiatry* 67 133–143. 10.1001/archgenpsychiatry.2009.193 20124113

[B55] Pearl S. B. Norton P. J. (2017). Transdiagnostic versus diagnosis specific cognitive behavioural therapies for anxiety: a meta-analysis. *J. Anxiety Disord.* 46 11–24. 10.1016/j.janxdis.2016.07.004 27466074

[B56] Pedersen B. K. Saltin B. (2015). Exercise as medicine - evidence for prescribing exercise as therapy in 26 different chronic diseases. *Scand. J. Med. Sci. Sports* 25(Suppl. 3), 1–72. 10.1111/sms.12581 26606383

[B57] Perez-Lopez F. R. Martinez-Dominguez S. J. Lajusticia H. Chedraui P. (2017). Effects of programmed exercise on depressive symptoms in midlife and older women: a meta-analysis of randomized controlled trials. *Maturitas* 106 38–47. 10.1016/j.maturitas.2017.09.001 29150165

[B58] Plummer P. Zukowski L. A. Giuliani C. Hall A. M. Zurakowski D. (2015). Effects of physical exercise interventions on gait-related dual-task interference in older adults: a systematic review and meta-analysis. *Gerontology* 62 94–117. 10.1159/000371577 25721432

[B59] Poyatos-Leon R. Garcia-Hermoso A. Sanabria-Martinez G. Alvarez-Bueno C. Cavero-Redondo I. Martinez-Vizcaino V. (2017). Effects of exercise-based interventions on postpartum depression: a meta-analysis of randomized controlled trials. *Birth* 44 200–208. 10.1111/birt.12294 28589648

[B60] Pritchett R. V. Daley A. J. Jolly K. (2017). Does aerobic exercise reduce postpartum depressive symptoms? a systematic review and meta-analysis. *Br. J. Gen. Pract.* 67 e684–e691. 10.3399/bjgp17X692525 28855163 PMC5604832

[B61] Razazian N. Yavari Z. Farnia V. Azizi A. Kordavani L. Bahmani D. S. (2016). Exercising impacts on fatigue, depression, and paresthesia in female patients with multiple sclerosis. *Med. Sci. Sports Exerc.* 48 796–803. 10.1249/MSS.0000000000000834 26656775

[B62] Rosenbaum S. Tiedemann A. Sherrington C. Curtis J. Ward P. B. (2014). Physical activity interventions for people with mental illness: a systematic review and meta-analysis. *J. Clin. Psychiatry* 75 964–974. 10.4088/JCP.13r08765 24813261

[B63] Rosenbaum S. Vancampfort D. Steel Z. Newby J. Ward P. B. Stubbs B. (2015). Physical activity in the treatment of Post-traumatic stress disorder: a systematic review and meta-analysis. *Psychiatry Res.* 230 130–136. 10.1016/j.psychres.2015.10.017 26500072

[B64] Roy T. Lloyd C. E. (2012). Epidemiology of depression and diabetes: a systematic review. *J. Affect. Disord.* 142(Suppl.), S8–S21. 10.1016/S0165-0327(12)70004-6 23062861

[B65] Salehi I. Hosseini S. M. Haghighi M. Jahangard L. Bajoghli H. Gerber M. (2016). Electroconvulsive therapy (ECT) and aerobic exercise training (AET) increased plasma BDNF and ameliorated depressive symptoms in patients suffering from major depressive disorder. *J. Psychiatr. Res.* 76 1–8. 10.1016/j.jpsychires.2016.01.012 26859236

[B66] Saligheh M. Hackett D. Boyce P. Cobley S. (2017). Can exercise or physical activity help improve postnatal depression and weight loss? A systematic review. *Arch. Womens Mental Health* 20 595–611. 10.1007/s00737-017-0750-9 28702773

[B67] Sartori C. R. Vieira A. S. Ferrari E. M. Langone F. Tongiorgi E. Parada C. A. (2011). The antidepressive effect of the physical exercise correlates with increased levels of mature BDNF, and proBDNF proteolytic cleavage-related genes, p11 and tPA. *Neuroscience* 180 9–18. 10.1016/j.neuroscience.2011.02.055 21371535

[B68] Schuch F. Vancampfort D. Firth J. Rosenbaum S. Ward P. Reichert T. (2017). Physical activity and sedentary behavior in people with major depressive disorder: a systematic review and meta-analysis. *J. Affect. Disord.* 210 139–150. 10.1016/j.jad.2016.10.050 28033521

[B69] Schuch F. B. Morres I. D. Ekkekakis P. Rosenbaum S. Stubbs B. (2016). A critical review of exercise as a treatment for clinically depressed adults: time to get pragmatic. *Acta Neuropsychiatr.* 29 65–71. 10.1017/neu.2016.21 27145824

[B70] Shors T. J. Millon E. M. Chang H. Y. Olson R. L. Alderman B. L. (2017). Do sex differences in rumination explain sex differences in depression? *J. Neurosci. Res.* 95 711–718. 10.1002/jnr.23976 27870434

[B71] Soo H. Burney S. Basten C. (2009). The role of rumination in affective distress in people with a chronic physical illness: a review of the literature and theoretical formulation. *J. Health Psychol.* 14 956–966. 10.1177/1359105309341204 19786522

[B72] Spinhoven P. Drost J. van Hemert B. Penninx B. W. (2015). Common rather than unique aspects of repetitive negative thinking are related to depressive and anxiety disorders and symptoms. *J. Anxiety Disord.* 33 45–52. 10.1016/j.janxdis.2015.05.001 26004746

[B73] Stepankova L. Kralikova E. Zvolska K. Pankova A. Ovesna P. Blaha M. (2016). Depression and smoking cessation: evidence from a smoking cessation clinic with 1-year follow-up. *Ann. Behav. Med.* 51 454–463. 10.1007/s12160-016-9869-6 28035641 PMC5440483

[B74] Stubbs B. Rosenbaum S. Vancampfort D. Ward P. B. Schuch F. B. (2016). Exercise improves cardiorespiratory fitness in people with depression: a meta-analysis of randomized control trials. *J. Affect. Disord.* 190 249–253. 10.1016/j.jad.2015.10.010 26523669

[B75] Stubbs B. Vancampfort D. Rosenbaum S. Firth J. Cosco T. Veronese N. (2017). An examination of the anxiolytic effects of exercise for people with anxiety and stress-related disorders: a meta-analysis. *Psychiatry Res.* 249 102–108. 10.1016/j.psychres.2016.12.020 28088704

[B76] Sukhato K. Lotrakul M. Dellow A. Ittasakul P. Thakkinstian A. Anothaisintawee T. (2017). Efficacy of home-based non-pharmacological interventions for treating depression: a systematic review and network meta-analysis of randomised controlled trials. *BMJ Open* 7:e014499. 10.1136/bmjopen-2016-014499 28706086 PMC5734422

[B77] Tang F. Wang G. Lian Y. (2017). Association between anxiety and metabolic syndrome: a systematic review and meta-analysis of epidemiological studies. *Psychoneuroendocrinology* 77 112–121. 10.1016/j.psyneuen.2016.11.025 28027497

[B78] Tarpada S. P. Morris M. T. (2017). Physical activity diminishes symptomatic decline in chronic schizophrenia: a systematic review. *Psychopharmacol. Bull.* 47 41–52. 28936008 10.64719/pb.4555 PMC5601086

[B79] Teicher M. H. Samson J. A. (2016). Annual research review: enduring neurobiological effects of childhood abuse and neglect. *J. Child Psychol. Psychiatry* 57 241–266. 10.1111/jcpp.12507 26831814 PMC4760853

[B80] Ussher M. H. Taylor A. H. Faulkner G. E. (2014). Exercise interventions for smoking cessation. *Cochrane Database Syst. Rev.* 8:CD002295. 10.1002/14651858.CD002295.pub5 18843632

[B81] Vancampfort D. De Hert M. Stubbs B. Soundy A. De Herdt A. Detraux J. (2015a). A systematic review of physical activity correlates in alcohol use disorders. *Arch. Psychiatr. Nurs.* 29 196–201. 10.1016/j.apnu.2014.08.006 26165972

[B82] Vancampfort D. Firth J. Schuch F. B. Rosenbaum S. Mugisha J. Hallgren M. (2017a). Sedentary behavior and physical activity levels in people with schizophrenia, bipolar disorder and major depressive disorder: a global systematic review and meta-analysis. *World Psychiatry* 16 308–315. 10.1002/wps.20458 28941119 PMC5608847

[B83] Vancampfort D. Stubbs B. Hallgren M. Veronese N. Mugisha J. Probst M. (2017b). Correlates of physical activity among community-dwelling individuals aged 65 years or older with anxiety in six low- and middle-income countries. *Int. Psychogeriatr.* 10.1017/S1041610217002216 [Epub ahead of print]. 29113616

[B84] Vancampfort D. Stubbs B. Herring M. P. Hallgren M. Koyanagi A. (2017c). Sedentary behavior and anxiety: association and influential factors among 42,469 community-dwelling adults in six low- and middle-income countries. *Gen. Hosp. Psychiatry* 50 26–32. 10.1016/j.genhosppsych.2017.09.006 28987919

[B85] Vancampfort D. Stubbs B. Koyanagi A. (2017d). Physical activity correlates in people with anxiety: Data from 46 low- and middle-income countries. *Gen. Hosp. Psychiatry* 49 26–31. 10.1016/j.genhosppsych.2017.04.007 29122146

[B86] Vancampfort D. Stubbs B. Mitchell A. J. De Hert M. Wampers M. Ward P. B. (2015b). Risk of metabolic syndrome and its components in people with schizophrenia and related psychotic disorders, bipolar disorder and major depressive disorder: a systematic review and meta-analysis. *World Psychiatry* 14 339–347. 10.1002/wps.20252 26407790 PMC4592657

[B87] Vancampfort D. Stubbs B. Richards J. Ward P. B. Firth J. Schuch F. B. (2016). Physical fitness in people with posttraumatic stress disorder: a systematic review. *Disabil. Rehabil.* 39 2461–2467. 10.1080/09638288.2016.1226412 27628485

[B88] Williams J. M. Ziedonis D. (2004). Addressing tobacco among individuals with a mental illness or an addiction. *Addict. Behav.* 29 1067–1083. 10.1016/j.addbeh.2004.03.009 15236808

[B89] Wu P. L. Lee M. Huang T. T. (2017). Effectiveness of physical activity on patients with depression and Parkinson’s disease: a systematic review. *PLoS One* 12:e0181515. 10.1371/journal.pone.0181515 28749970 PMC5531507

